# Hub Genes and Key Pathway Identification in Colorectal Cancer Based on Bioinformatic Analysis

**DOI:** 10.1155/2019/1545680

**Published:** 2019-11-06

**Authors:** Jian Lv, Lili Li

**Affiliations:** Central Laboratory, Renmin Hospital of Wuhan University, Wuhan 430060, China

## Abstract

Colorectal cancer (CRC) is one of the most common malignant tumors. The aim of the present study was to identify key genes and pathways to improve the understanding of the mechanism of CRC. GSE87211, including 203 CRC samples and 160 control samples, was screened to identify differentially expressed genes (DEGs). In total, 853 DEGs were obtained, including 363 upregulated genes and 490 downregulated genes. Gene Ontology (GO) and Kyoto Encyclopedia of Genes and Genomes (KEGG) analysis of DEGs were performed to obtain enrichment datasets. GO analysis showed that DEGs were significantly enriched in the extracellular region, cell-cell signaling, hormone activity, and cytokine activity. KEGG pathway analysis revealed that the DEGs were mainly enriched in the cytokine-cytokine receptor interaction, drug metabolism, androgen and estrogen metabolism, and neuroactive ligand-receptor interaction. The Protein-Protein Interaction (PPI) network of DEGs was constructed by using Search Tool for the Retrieval of Interacting Genes (STRING). The app MCODE plugged in Cytoscape was used to explore the key modules involved in disease development. 43 key genes involved in the top two modules were identified. Six hub genes (CXCL_2_, CXCL_3_, PTGDR2, GRP, CXCL_11_, and AGTR1) were statistically associated with patient overall survival or disease-free survival. The functions of six hub genes were mainly related to the hormone and chemokine activities. In conclusion, the present study may help understand the molecular mechanisms of CRC development.

## 1. Introduction

Colorectal cancer (CRC) is one of the leading causes of malignant tumors [1, 2]. The incidence rate of CRC is high, which seriously affects the patient's health. Sigmoidoscopy has become an effective surgery for treating CRC; however, it is associated with the disadvantages of bleeding, perforation [3], and the low prognosis rate [4, 5]. Our understanding of the occurrence and development mechanism of CRC has been greatly improved; however, the cause and potential molecular mechanism of CRC are still unclear [6, 7]. Therefore, it is necessary to identify molecular hub genes and key pathways for understanding the molecular mechanism and discovering potential biomarkers for CRC.

At present, microarray technology is widely used in molecular mechanism exploration and has a wide range of application in molecular biology. It offers an efficient method for systematically screening tumor-related genes and identifying their regulatory mechanisms with bioinformatics [8, 9]. The hub genes are the highly connected nodes in the PPI network, which have high probabilities of engaging in essential biological regulation [10], and have been reported in various types of cancer. The Protein-Protein Interaction (PPI) network and hub genes analysis are used for revealing crucial biological processes [11], which provide efficient approaches for discovering key molecular mechanisms in cancer biology.

In this study, 853 differentially expressed genes (DEGs) were obtained by screening gene expression microarray dataset GSE87211, which includes 203 CRC samples and 160 control samples. The biological functions, signal pathway enrichment, and PPI network were used to establish the characterization of the DEGs for understanding the molecular mechanism underlying CRC. It might also provide new insights for the study of potential biomarkers of CRC.

## 2. Materials and Methods

### 2.1. Gene Expression Microarray Data Acquisition

The NCBI Gene Expression Omnibus (GEO, http://www.ncbi.nlm.nih.gov/geo) database is a public functional genomics database with high-throughput gene expression data, chips, and microarrays. GSE87211 [12] was downloaded from GEO. The datasets were based on the GPL13497 platform (Agilent-026652 Whole Human Genome Microarray 4 × 44 K v2). GSE87211 contains 203 CRC samples and 160 control samples.

### 2.2. Identification of DEGs

The DEGs were screened using linear models for microarray data (limma) package in R. Probe sets without corresponding gene symbols were removed, and they were further converted into the corresponding gene symbol according to the annotation information. The mean value of multiple probes for the same gene was calculated. |logFC(fold change)| > 2 and Adj. *P* value < 0.01 were considered as the threshold to identify the DEGs.

### 2.3. GO and KEGG Enrichment Analyses of DEGs

The Database for Annotation, Visualization, and Integrated Discovery (DAVID, http://david.ncifcrf.gov) (version 6.8) is an online functional annotation tool to provide a comprehensive understanding of biological information of genes and proteins, including biological process (BP), cellular components (CC), and molecular function (MF). Kyoto Encyclopedia of Genes and Genomes (KEGG) pathway enrichment was further annotated and viewed online by KEGG Orthology-Based Annotation System (KOBAS, http://www.kobas.cbi.pku.edu.cn) (version 3.0). *P* value < 0.05 was considered as the threshold.

### 2.4. PPI Network Construction and MCODE Analysis

The PPI network of DEGs was predicted using the online database Search Tool for the Retrieval of Interacting Genes (STRING, http://string-db.org) (version 11.0). The minimum required interaction score was set to 0.09. The protein nodes that have no interaction with other proteins were removed. Analyzing the functional interactions between proteins may provide insights into the biological mechanisms of action. Key modules and hub genes were further analyzed and visualized with app MCODE plugged in Cytoscape. The top two modules were displayed to show the density of nodes by STRING. The criteria for selection of key genes were as follows: MCODE scores >5.

### 2.5. Survival Analysis of Hub Genes

The correlation between the key genes and the survival time of CRC patients were analyzed through the survival function in R package by using the clinical information from GSE87211 and TCGA datasets. The overall survival and disease-free survival analyses of each hub gene were performed in Gene Expression Profiling Interactive Analysis (GEPIA, http://gepia.cancer-pku.cn/index.html) [13]. The RNA expression level of hub genes between CRC samples and control samples was visualized by GEPIA based on the integration of GTEx and TCGA projects in a standard processing pipeline. The protein expression level of hub genes was analyzed using the Human Protein Atlas database.

## 3. Results and Discussion

### 3.1. Identification of DEGs in CRC

**Figure 1:**
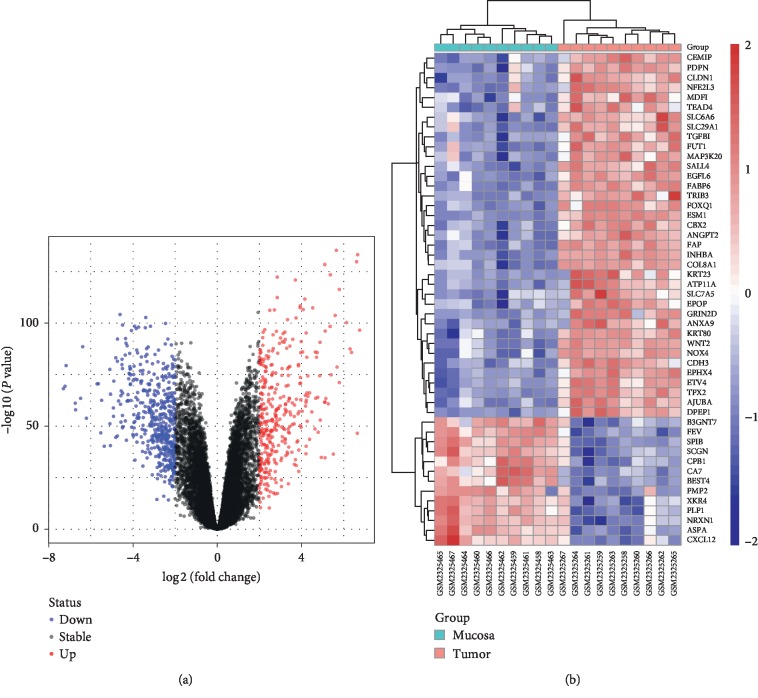
DEGs from the microarray dataset GSE87211. (a) 853 DEGs are shown in a volcano plot. 363 upregulated genes are shown in red, and 490 downregulated genes are shown in blue. (b) Heatmap of the top 50 most significant DEGs. 10 CRC samples and 10 control samples were randomly selected. Red denotes upregulated genes, and blue represents downregulated genes. The green and red bars represent normal and tumor groups, respectively. DEGs were identified with a classical *t*-test with cutoff value of |logFC| > 2 and Adj. *P* value < 0.01.

The gene expression profile of GSE87211, including 203 CRC samples and 160 control samples, was downloaded from the GEO database. The median value of each sample was normalized (Supplementary [Supplementary-material supplementary-material-1]). 853 DEGs were identified including 363 upregulated genes and 490 downregulated genes, shown in the volcano plot (Figure 1(a)). Randomly selected 10 CRC and 10 control samples were clustered together, respectively, according to the expression level of top 50 significant DEGs shown in the heatmap (Figure 1(b)).

### 3.2. GO and KEGG Enrichment Analyses of DEGs

**Figure 2:**
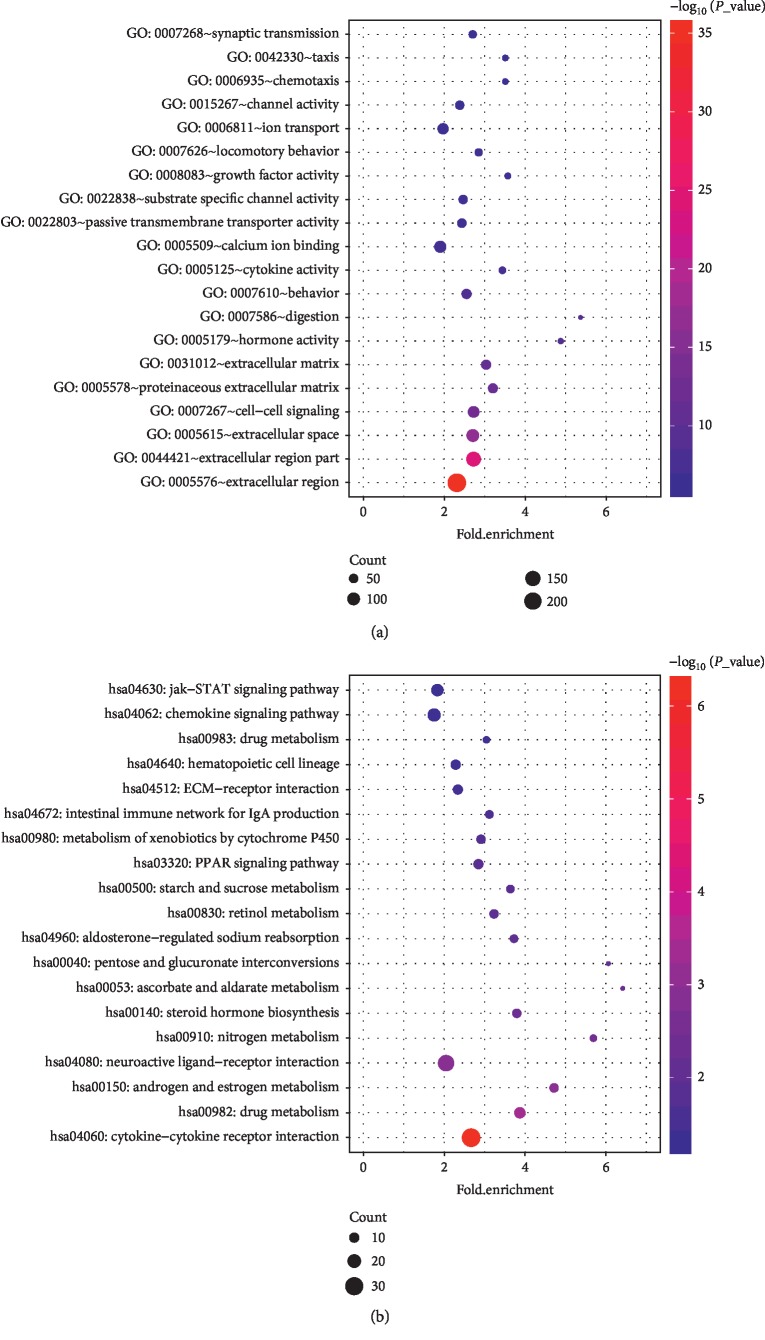
GO and KEGG enrichment analyses of DEGs shown in the bubble plot. (a) The top 20 enriched terms of GO analysis. Cutoff value is *P* value < 0.05. (b) The top 19 enriched terms of the KEGG pathway. Cutoff value is *P* value < 0.05. The size of dots indicates the count of DEGs enriched under each term. *P* value is represented by the colour of the dot.

To figure out the functions of DEGs, GO analysis was performed with DAVID tool. The DEGs were classified into three functional groups: biological processes (BP), cell component (CC), and molecular function (MF). GO analysis results showed that changes in BP were significantly enriched in digestion, behavior, locomotory behavior, ion transport, chemotaxis, taxis, synaptic transmission; changes in CC were mainly enriched in the extracellular region, extracellular region part, extracellular space, cell-cell signaling, proteinaceous extracellular matrix, and extracellular matrix; and changes in MF were mainly enriched in hormone activity, cytokine activity, calcium ion binding, passive transmembrane transporter activity, substrate-specific channel activity, growth factor activity, and channel activity (Figure 2(a)).

KEGG pathway analysis by KOBAS revealed that the DEGs were mainly enriched in cytokine-cytokine receptor interaction, drug metabolism, androgen and estrogen metabolism, neuroactive ligand-receptor interaction, nitrogen metabolism, steroid hormone biosynthesis, and so on (Figure 2(b)). From GO and KEGG results, we could anticipate that functions related to hormone activity, cytokine activity, and cytokine-cytokine receptor interaction might play important role in the progress of CRC.

### 3.3. PPI Network Construction and Key Genes Selection

**Figure 3:**
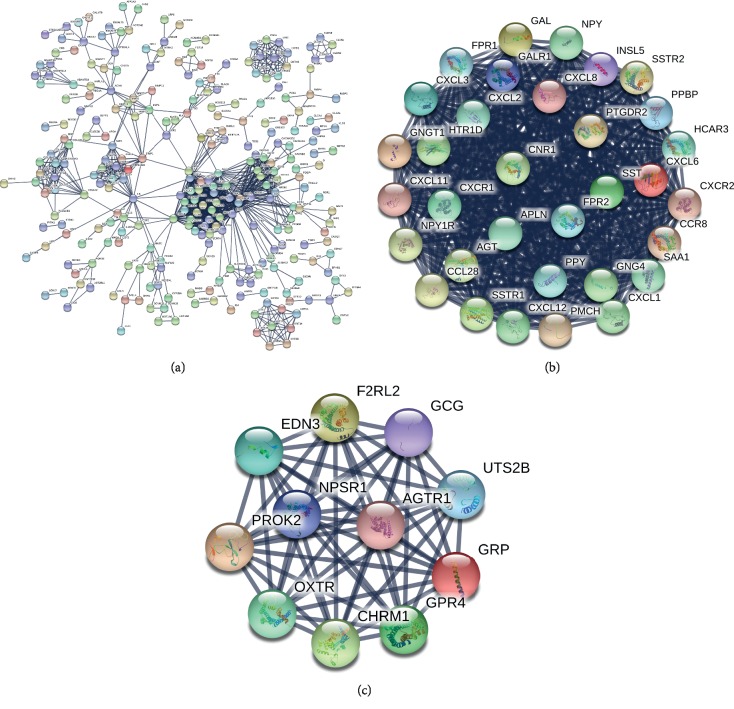
PPI network analysis. (a) PPI network with 279 nodes and 1201 edges was constructed using the highest stringent minimum required interaction score of 0.09 by STRING. (b) Module 1 consisted of 32 nodes and 473 edges. (c) Module 2 consisted of 11 nodes and 55 edges.

To discover the key gene, PPI network with 279 nodes and 1201 edges was constructed with the highest stringent minimum required interaction score of 0.09 (Figure 3(a)). A total of 43 genes in the top two modules were identified as key genes with score ≥5. 32 key genes in Module 1 and 11 key genes in Module 2 are shown as the density of nodes in Figures 3(b) and 3(c) (Table 1).

### 3.4. Hub Genes Selection and Validation

**Figure 4:**
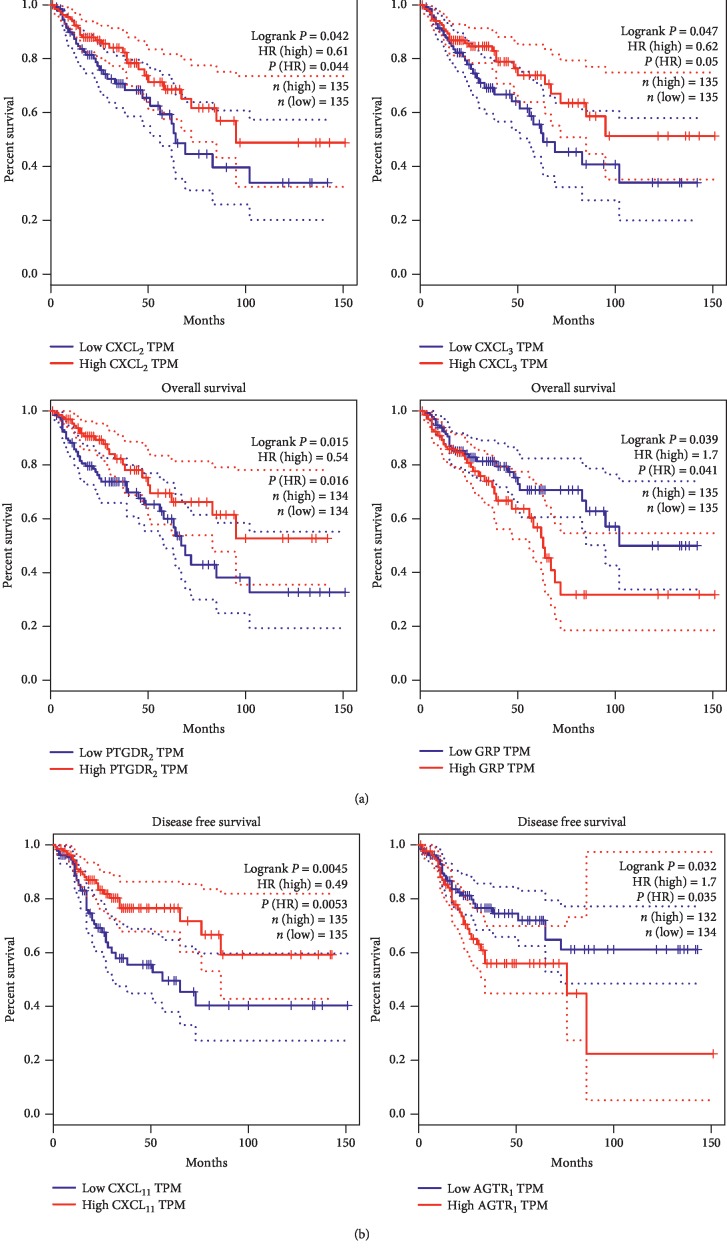
Survival analyses of six hub genes (CXCL_2_, CXCL_3_, PTGDR2, GRP, CXCL_11_, and AGTR1) using the GEPIA online platform. (a) The overall survival analyses of CXCL_2_, CXCL_3_, PTGDR2, and GRP. (b) The disease-free survival analyses of CXCL_11_ and AGTR1.

To select the hub genes from 43 key genes, overall survival analysis was firstly performed using clinical data obtained from the microarray dataset GSE87211. It showed that none of the key genes had a statistically significant relation to overall survival (Table 2). Then, the overall survival and disease-free survival analyses of each hub gene were performed by GEPIA using the TCGA dataset (Table 2). The alteration of CXCL_2_, CXCL_3_, PTGDR2, and GRP genes was statistically associated with a worse overall survival rate, whereas genes CXCL_11_ and AGTR1 were statistically associated with a worse disease-free survival rate (Table 2). Patients with a higher gene expression level of CXCL_2_, CXCL_3_, PTGDR2, and CXCL_11_ have a significantly better prognosis compared to those with lower expression level, while patients with lower expression of GRP and AGTR1 were shown better survival rates (Figure 4). The inconsistent overall survival results between GSE87211 and TCGA might be due to the limited size of the clinical samples.

**Figure 5:**
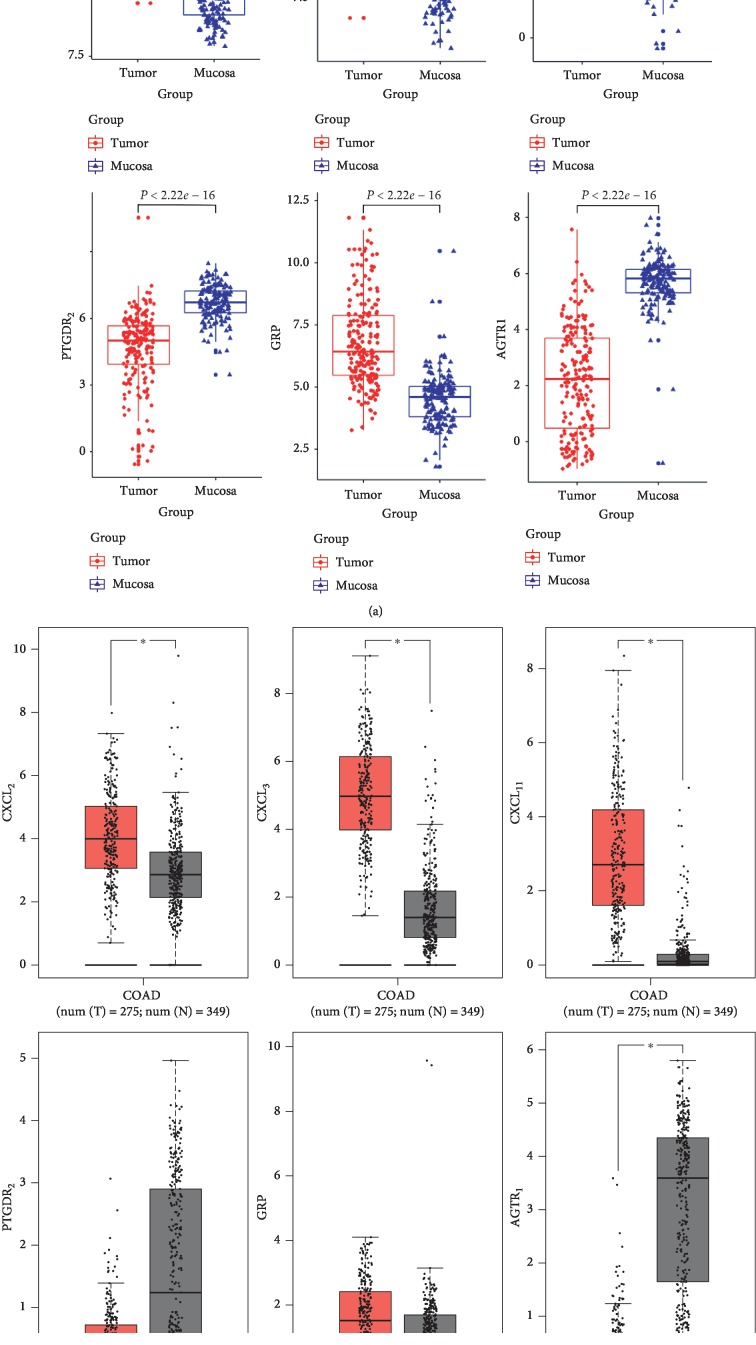
RNA expression level of six hub genes in CRC samples and control samples. Data were obtained from GSE87211 (a) and TCGA (red: tumor; gray: normal) (b). ^*∗*^*P* value <0.05. COAD: colon adenocarcinoma.

**Figure 6:**
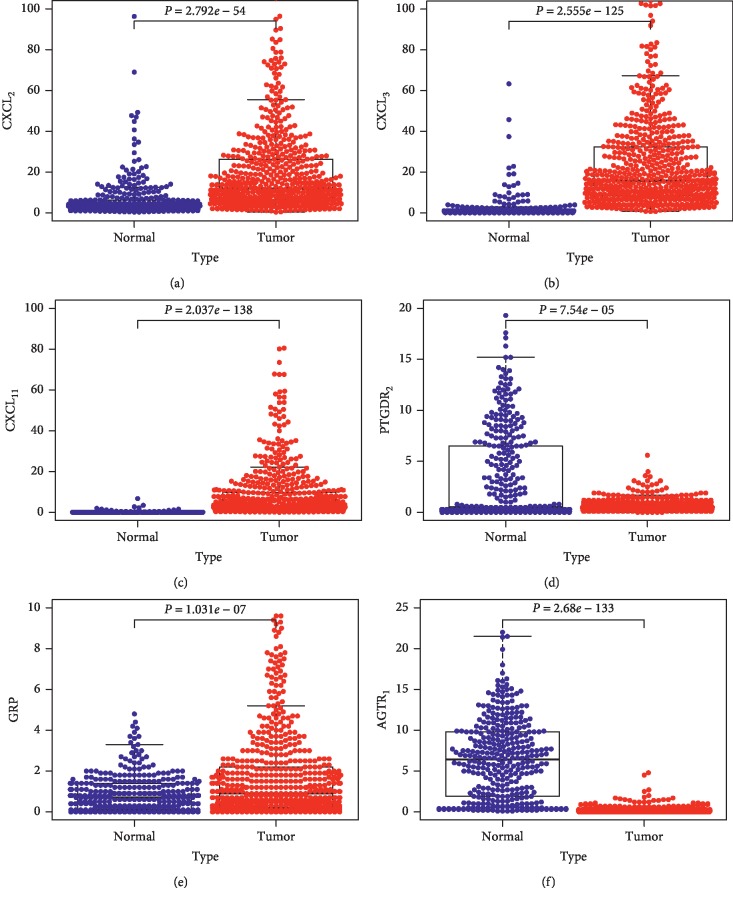
Protein expression level of hub genes in the Human Protein Atlas dataset.

RNA and protein expression levels of CXCL_2_, CXCL_3_, CXCL_11_, and AGTR1 genes were statistically different between the CRC and control samples, and these results were also consistent with the RNA expression level observed in microarray dataset GSE87211 (Figures 5 and 6). Furthermore, the RNA expression level of six hub genes was significantly different in various types of cancer and suggested that they played various functions in the progress of different cancers (Supplementary [Supplementary-material supplementary-material-1]).

## 4. Discussion

CRC is a disease caused by high modality. Gene mutations and abnormal expression have been demonstrated in the progression of CRC. Understanding the molecular mechanism of CRC is critically important for diagnosis and treatment. With the development of microarray and high-throughput sequencing, the alternative expression levels of thousands of genes could be simultaneously screened. Integrating and reanalyzing microarray data provide valuable information including hub genes, biological functions, and signaling pathways, which indicate new clues for the diagnosis and treatment of CRC.

In this study, we extracted the expression data of GSE87211 from the GEO dataset with high-quality data and clinical characters. The previous study reported that the microarray dataset GSE87211 was used for examining the expression level of genes potentially linked with the SNPs and studying the CRC risk loci by SNP array analysis [12]. However, no deep microarray analysis was shown in the previous study. In this study, we reanalyze the microarray data to explore the key genes involved in the molecular mechanism of CRC.

853 DEGs were identified, including 363 upregulated genes and 490 downregulated genes. It showed that DEGs were significantly enriched in terms of extracellular region, cell-cell signaling, cytokine-cytokine receptor interaction, drug metabolism, androgen and estrogen metabolism, neuroactive ligand-receptor interaction, and nitrogen metabolism. 43 key genes were identified with potential clinical value. Six hub genes (CXCL_2_, CXCL_3_, PTGDR2, GRP, CXCL_11_, and AGTR1) showed statistically different expression between the CRC and control samples and statistical correlation with the prognosis of CRC patients.

CXCL_11_ and CXCL_2_ are belonged to the chemokine's superfamily, which are group of small secreted proteins. Chemokines could bind to G protein-coupled transmembrane receptors on target cells and recruit cells of the immune system to a site of infection. CXCR3 is G protein-coupled transmembrane receptor. Together, they play fundamental roles in the development, homeostasis, and function of the immune system. CXCL_11_, induced by IFN-c and IFN-b, has a high affinity with CXCR3. The CXCL_11_/CXCR3 axis regulates the differentiation of naive T cells to T helper 1 cells and regulates immune cell migration, differentiation, and activation [14]. The previous study had demonstrated that chemokines could exert antitumor effects via recruiting T cells, enhancing immune responses, and suppressing tumor-associated angiogenesis [15]. Chimeric molecules CXCL_10_ and CXCL_11_ had an impressive antitumor efficacy [16]. However, studies also indicated that CXCL_11_ played important roles in promoting the chemotaxis activity of TAM, which was related to the poor prognosis of colorectal cancer. It had been reported that the calculated score based on the expression of CXCL_11_, CXCL_9_, and CXCL_10_ could stratify nonmetastatic clear-cell renal cell carcinoma (ccRCC) patients into different risk subgroups [17]. CXCR3 was upregulated and played a predominant role in the tumorigenicity of prostate cancer. It showed that overexpression of CXCR3 stimulated the proliferation and migration of cancer cells *in vitro* and *in vivo*, which are related to the progression of malignancies [18]. Secretome study in breast cancer cell lines revealed that CXCR3 was highly upregulated in aggressive cancer cells and revealed a functional role of CXCR3 as a potential target for cancer therapy [19].

CXCL_2_ (C-X-C motif chemokine ligand 2) significantly enhanced the migration and invasion ability of hepatocellular carcinoma cells (HCCs). CXCL_2_ overexpression profoundly attenuated HCC proliferation and growth and induced apoptosis *in vivo* by negatively regulating the cell cycle via the ERK1/2 signaling pathway [20]. CXCL_2_ is expressed at sites of inflammation and may suppress hematopoietic progenitor cell proliferation. However, CXCL_2_ has also been reported to act as an oncogene. The methylation status of CXCL_2_ was significantly different between normal and hepatocellular carcinoma tissues. Tissues with higher CXCL_2_ expression showed significantly more numbers of tumors, indicating that the regulation mechanism may be controlled by CXCL_2_ methylation [21]. CXCL_2_ knockdown results showed reduced expression of cancer stem cell proteins, cyclins, and EMT markers, mediating through G*α*i-2 and G*α*q/11 to promote tumorigenesis [22].

PTGDR2, also named CRTH2, is a prostaglandin D2 receptor and preferentially expresses in CD4^+^ effector T helper 2 cells. PTGDR2 is the receptor of PGD2. Knockdown of PTGDR2 and PGD2 expression in cancer stem cells (CSCs) resulted in enhanced expression of CSC markers and self-renewal ability. PGD2 inhibited tumor growth, incidence rate, and mesenteric metastasis *in vivo*. Further study showed that the expression of PTGDR2 reversed these effects, indicating a novel function of PGD2/PTGDR2 signaling on CSC regulation and tumorigenesis in gastric cancer [23]. Numbers of research about PTGDR2 focused on studying colon inflammation-related diseases. The high expression level of PTGDR2 was predominantly observed in the mild inflammation in ulcerative colitis patients [24]. PTGDR2 also played a proinflammatory role in the TNBS-induced colitis model. Antagonism of PTGDR2 had been shown to promote antiallergic and anti-inflammatory effects in Crohn's disease [25]. PTGDR2, a transmembrane protein, may have more potential as cancer targets.

GRP, gastrin-releasing peptide, mainly regulates numerous functions of the gastrointestinal and central nervous systems and plays an important role in human various cancers. GRP expression may be a predictive of aggressive tumor biomarkers for stratifying stages of colorectal cancer [26]. Downregulation of the GRP reduced the numbers of cancer stem cells *in vitro* and further abolished tumor development in SCID mice [27]. In small-cell lung cancer (SCLC), the high expression level of GRP was related to high disease burden and negative prognostic signature might be used as a potential diagnosis biomarker [28].

AGTR1, angiotensin II receptor type 1, is a potent vasopressor hormone and acts as an important effector controlling blood pressure and volume in the cardiovascular system. DNA methylation of AGTR1 might be a performing candidate biomarker, screened by paired normal and CRC stool samples [29]. AGTR1 mediated cell movement and promoted lymph node metastasis by activating the FAK/RhoA pathway in early-stage breast cancer [30]. AGTR1 hypermethylation is a promising biomarker in lung squamous cell carcinoma detection and diagnosis [31].

## 5. Conclusions

In conclusion, our present study performed a bioinformatic analysis of DEGs between paired normal and CRC to obtain hub genes, providing certain key pathway in the occurrence and progress of CRC. The real function of these hub genes needs to be explored further to determine the clinical and biological mechanism of CRC.

## Figures and Tables

**Table 1:** Scores and the included key genes in the top two modules.

Module	Score	Gene symbol
1	15.5	CXCL_11_, SSTR1, SSTR2, CXCL_12_, HCAR3, SST, APLN, CXCL_8_, CXCR2, PPY, NPY, PPBP, SAA1, PMCH, GAL, CXCR1, NPY1R, CCL_23_, CXCL_6_, CCL_28_, FPR2, CXCL_1_, CXCL_2_, CXCL_3_, HTR1D, GALR1, CNR1, AGT, FPR1, PTGDR2, CCR8, INSL5
2	5	F2RL2, GCC, GRP, OXTR, GPR4, NPSR1, UTS2B, PROK2, AGTR1, EDN3, CHRM1

**Table 2:** The overall survival and disease-free survival analyses of each key gene were performed using clinical data from GSE87211 or TCGA database. ^*∗*^*P* value <  0.05.

MCODE	Gene symbol	Overall survival in GSE87211 (*P* value)	Overall survival in TCGA (*P* value)	Disease-free survival in TCGA (*P* value)
1	CXCL_11_	0.48	0.54	0.0045^*∗*^
1	SSTR1	0.58	0.54	0.73
1	SSTR2	0.44	0.74	0.48
1	CXCL_12_	0.32	0.68	0.46
1	HCAR3	0.92	0.85	0.78
1	SST	0.42	0.75	0.94
1	APLN	0.38	0.4	0.26
1	CXCL_8_	0.81	0.05	0.41
1	CXCR2	0.42	0.56	0.57
1	PPY	0.61	NA	NA
1	NPY	0.39	0.31	0.12
1	PPBP	0.59	0.22	0.084
1	SAA1	0.41	0.98	0.53
1	PMCH	0.42	0.6	0.17
1	GAL	0.67	0.23	0.83
1	CXCR1	0.94	0.46	0.77
1	NPY1R	0.74	0.71	0.75
1	CCL_23_	0.44	0.79	0.98
1	CXCL_6_	0.42	0.44	0.35
1	CCL_28_	0.62	0.37	0.9
1	FPR2	0.57	0.45	0.87
1	CXCL_1_	0.2	0.13	0.34
1	CXCL_2_	0.064	0.042^*∗*^	0.49
1	CXCL_3_	0.26	0.047^*∗*^	0.46
1	HTR1D	0.71	0.19	0.096
1	GALR1	0.69	NA	NA
1	CNR1	0.82	1	0.27
1	AGT	0.89	0.073	0.11
1	FPR1	0.48	0.59	0.89
1	PTGDR2	0.81	0.015^*∗*^	0.36
1	CCR8	0.55	0.37	0.7
1	INSL5	0.87	NA	NA
2	F2RL2	0.39	0.16	0.97
2	GCG	0.51	0.056	0.38
2	GRP	0.7	0.039^*∗*^	0.28
2	OXTR	0.75	0.17	0.65
2	GPR4	0.51	0.65	0.4
2	NPSR1	0.15	0.22	0.73
2	UTS2B	0.15	0.63	0.88
2	PROK2	0.87	0.61	0.98
2	AGTR1	0.82	0.05	0.032^*∗*^
2	EDN3	0.89	0.064	0.46
2	CHRM1	0.82	0.23	0.33

## Data Availability

The data of GSE87211 were downloaded from the NCBI Gene Expression Omnibus database (GEO, http://www.ncbi.nlm.nih.gov/geo).

## Supplementary Materials

Supplementary Materials Supplementary Figure 1: boxplot of the distribution of each sample in GSE87211. NormalizeBetweenArrays function in limma package was used for normalization. (a) The boxplot before normalization and (b) the normalized boxplot. Supplementary Figure 2: the RNA expression level of six hub genes in various types of cancer in the TCGA dataset analyzed by GEPIA. T indicates tumor, and N indicates normal.
