# Supplementary material for: Hub Genes and Key Pathway Identification in Colorectal Cancer Based on Bioinformatic Analysis
Source: Biomed Res Int. 2019 Nov 6;2019:1545680. doi: 10.1155/2019/1545680 (PMC6874977; doi:10.1155/2019/1545680)
Supplement: Supplementary Materials — Supplementary Figure 1: boxplot of the distribution of each sample in GSE87211. NormalizeBetweenArrays function in limma package was used for normalization. (a) The boxplot before normalization and (b) the normalized boxplot. Supplementary Figure 2: the RNA expression level of six hub genes in various types of cancer in the TCGA dataset analyzed by GEPIA. T indicates tumor, and N indicates normal. [file 1545680.f1.pdf]

Supplementary Figures

a

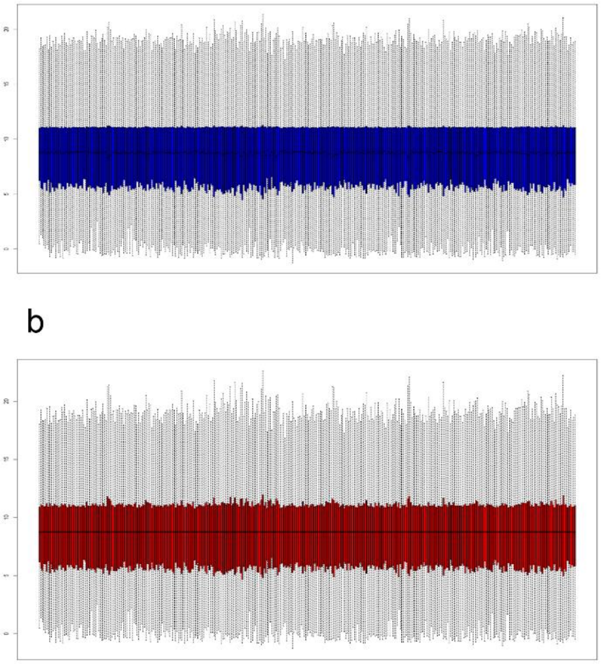

Supplementary Figure 1. Boxplot of the distribution of each sample in GSE87211. NormalizeBetweenArrays function in limma package was used for normalization. (a) The boxplot before normalization, (b) The normalized boxplot.

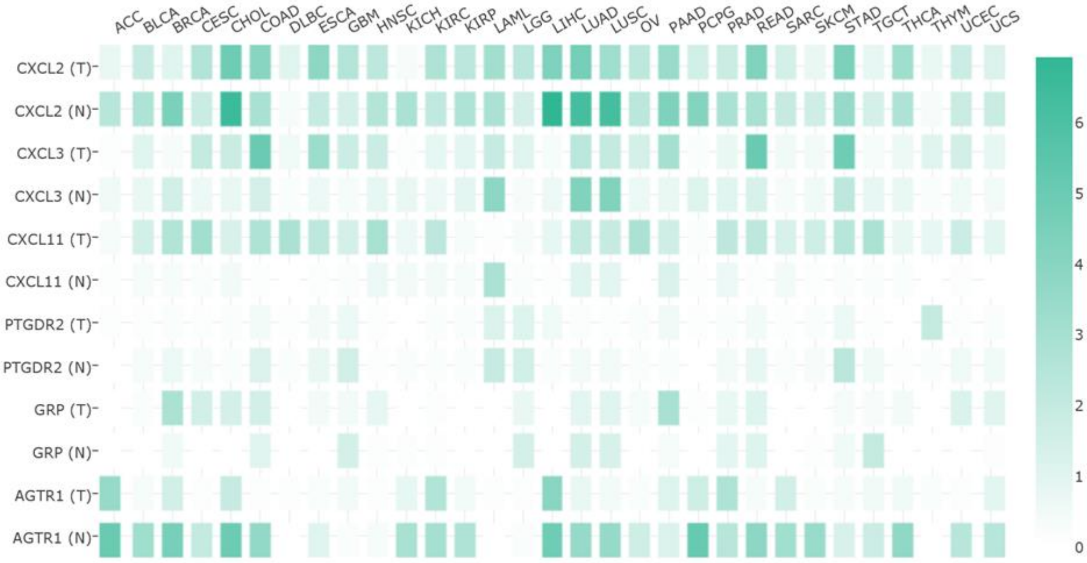

Supplementary Figure 2. The RNA expression level of six hub genes in various cancer in the TCGA dataset analyzed by GEPIA. T indicates tumor, and N indicates normal.
